# Succeeding in deactivating: associations of hair zinc levels with functional and structural neural mechanisms

**DOI:** 10.1038/s41598-020-69277-4

**Published:** 2020-07-23

**Authors:** Hikaru Takeuchi, Yasuyuki Taki, Rui Nouchi, Ryoichi Yokoyama, Yuka Kotozaki, Seishu Nakagawa, Atsushi Sekiguchi, Kunio Iizuka, Sugiko Hanawa, Tsuyoshi Araki, Carlos Makoto Miyauchi, Kohei Sakaki, Takayuki Nozawa, Shigeyuki Ikeda, Susum Yokota, Daniele Magistro, Yuko Sassa, Ryuta Kawashima

**Affiliations:** 1 Division of Developmental Cognitive Neuroscience, Institute of Development, Aging and Cancer, Tohoku University, 4-1 Seiryo-cho, Aoba-ku, Sendai, 980-8575 Japan; 2 Division of Medical Neuroimaging Analysis, Department of Community Medical Supports, Tohoku Medical Megabank Organization, Tohoku University, Sendai, Japan; 3 Department of Radiology and Nuclear Medicine, Institute of Development, Aging and Cancer, Tohoku University, Sendai, Japan; 4 Creative Interdisciplinary Research Division, Frontier Research Institute for Interdisciplinary Science, Tohoku University, Sendai, Japan; 5 Human and Social Response Research Division, International Research Institute of Disaster Science, Tohoku University, Sendai, Japan; 6 Department of Advanced Brain Science, Institute of Development, Aging and Cancer, Tohoku University, Sendai, Japan; 7 School of Medicine, Kobe University, Kobe, Japan; 8 Division of Clinical Research, Medical-Industry Translational Research Center, Fukushima Medical University School of Medicine, Fukushima, Japan; 9 Department of Human Brain Science, Institute of Development, Aging and Cancer, Tohoku University, Sendai, Japan; 10 Division of Psychiatry, Tohoku Medical and Pharmaceutical University, Sendai, Japan; 11 Department of Behavioral Medicine, National Institute of Mental Health, National Center of Neurology and Psychiatry, Tokyo, Japan; 12 Department of Psychiatry, Tohoku University Graduate School of Medicine, Sendai, Japan; 13 Advantage Risk Management Co., Ltd., Tokyo, Japan; 14 Research Center for the Earth Inclusive Sensing Empathizing with Silent Voices, Tokyo Institute of Technology, Tokyo, Japan; 15 Department of Ubiquitous Sensing, Institute of Development, Aging and Cancer, Tohoku University, Sendai, Japan; 16 Division for Experimental Natural Science, Faculty of Arts and Science, Kyushu University, Fukuoka, Japan; 17 Department of Sport Science, School of Science and Technology, Nottingham Trent University, Nottingham, UK

**Keywords:** Neuroscience, Biomarkers

## Abstract

Zinc is a biologically essential element and involved in a wide range of cellular processes. Here, we investigated the associations of zinc levels in hair with brain activity during the n-back working memory task using functional magnetic resonance imaging, fractional anisotropy (FA) of diffusion tensor imaging, and cognitive differences in a study cohort of 924 healthy young adults. Our findings showed that greater hair zinc levels were associated with lower brain activity during working memory in extensive areas in the default mode network (i.e., greater task-induced deactivation) as well as greater FA in white matter areas near the hippocampus and posterior limbs of the internal capsule. These findings advance previous non-neuroimaging findings of zinc’s associations with excitability, excitability-associated disorders, and myelination.

## Introduction

Zinc is an essential element in both brain and systemic physiology, as it is a component of more than 300 enzymes and involved in a wide range of normal cellular processes^1^. However, dysregulated greater level of zinc is neurotoxic and can harm neural tissue. Therefore, either high or low zinc can be detrimental to neural tissues^2^.

One of zinc’s key roles in the brain is modulation of neuronal excitability. Zinc is associated with inhibition of excitatory and inhibitory receptors as well as modulation of glutamate and GABA release, but reduced brain zinc availability leads to excitability, such as epileptiform brain activity, which suggests that the dominant effect of zinc in the normal brain is to reduce excitability^3,4^. Consistent with this hypothesis, epileptic patients have been shown to have reduced zinc levels^5^. There is also evidence that greater zinc levels or zinc supplementation are associated with greater sleep quality and quantity; however, the modulation of neural excitability caused by zinc may underlie these findings^6^. An increased excitability/inhibition ratio is thought to play a key role in the pathology of autism and schizophrenia^7,8^, and interestingly, children with autism (but not adults were shown to have robustly low hair zinc levels in the huge sample^9^ and patients with schizophrenia were shown to have low hair zinc levels in multiple studies^10,11^ and they also show substantially reduced brain zinc levels^12^. Zinc deficits are also known to lead to socializing deficits^13^. Animal studies have shown that zinc deprivation leads to poor performance in attention and short-term memory tasks^2^.

Further, zinc is known to play specific roles in the hippocampus. Zinc is highly concentrated in the hippocampus, and zinc deprivation has been shown to result in deficits in hippocampal activation and abrogate long-term potentiation (LTP), reduce hippocampal neurogenesis, and increase neuronal apoptosis that destroys structural hippocampal plasticity^1,14,15^. Animal studies have shown that zinc is important for hippocampal memory function via the abovementioned hippocampal activity^15^.

Previous neuroimaging studies have shown that zinc supplementation leads to reduction in electroencephalogram (EEG) amplitude^5^. Despite zinc’s wide range of physiological roles and the behavioral deficits and pathologies associated with zinc deprivation, the associations of body zinc level with brain activity measured by functional magnetic resonance imaging (fMRI) and white matter structure remain unknown. The purpose of this study was thus to investigate these issues. To achieve this, we assessed a wide range of cognitive functions, fMRI brain activity during the n-back working memory task, fractional anisotropy (FA) of diffusion tensor imaging (DTI), and hair zinc levels in a large sample of young adults. We also utilized a wide range of cognitive measures to see if zinc’s previously reported effects were seen in our sample and to reveal the nature of cognitive correlates of hair zinc levels.

As summarized in previous studies and mostly reproduced below^16,17^, hair mineral analysis is often assumed to be the best indicator of mineral levels in the body and has been used in multiple fields^18^. Hair is a repository of all elements that enter the body, and mineral levels in hair reflect accumulation of mineral composition over several months to years^16^. And thus, hair mineral levels are not affected substantially by rapid fluctuation in mineral intake and show long-term stability^19^. These characteristics give hair mineral analysis advantages over other methods to measure mineral levels such as blood and urine analyses. Studies showed positive correlation between concentrations of basic elements in the hair and in the body^20,21^. However, it was also suggested that hair mineral analysis requires, sampling by trained personnel, with standardized pre-analytical and analytical procedures, using suitable and sensitive equipment were required to obtain comparable results^22^. And new analytic methods and good practice have improved the precision of hair mineral analysis^23^. Zinc supplementation robustly increases the hair zinc content compared with controls^24^. A recent systematic review confirmed that, in healthy individuals, hair zinc is a reliable biomarker of zinc status^25^. Another review suggested that hair zinc is a biomarker of nutritional value and that zinc deficiency may be the consequence of dietary unavailability, excessive zinc loss, or inherited metabolic disturbances^26^. Unlike serum zinc, hair zinc levels are more stable and unaffected by diurnal variation, prolonged fasting, meal consumption, and acute infection^27^. In addition, although the bioavailability of zinc is reduced by phytates in foods of vegetable origin^27^, studies have shown that diets with high phytate intakes or high phytate:zinc molar ratios are associated with lower hair zinc levels^28–31^.

We hypothesized that greater task-induced deactivation (TID) in the default mode network (DMN) during working memory would be associated with greater hair zinc levels. This hypothesis is based on findings that TID in the DMN is associated with brain excitability/inhibition underlain by glutamate and GABA^32^, which are in turn associated with body zinc levels as described above. Further, TID in the DMN particularly during the externally directed attention demanding tasks has been used as measures to reflect brain excitability or failure to deactivate e.g.,^33,34^. Based on the specific associations of zinc with hippocampal activity, we predicted that greater hippocampal activity would be associated with greater hair zinc levels. We also hypothesized that greater FA would be associated with greater hair zinc levels based on evidence that zinc is essential for myelination^35^.

## Methods

### Subjects

The present study, which is a part of an ongoing project to investigate associations between brain imaging, cognitive function, and aging, included 924 healthy right-handed individuals (563 men and 361 women) from whom the data necessary for whole-brain analyses involving Zn levels were collected. The mean subject age was 20.7 years (standard deviation (SD), 1.8; age range: 18–27 years). Written informed consent was obtained from adult subjects. For nonadult subjects (age < 20 y.o.), written informed consent was obtained from their parents (guardians). This study was approved by the Ethics Committee of Tohoku University. For more detailed subject information, see Supplemental Methods.

### Hair acquisition and hair mineral analysis

Scalp hair samples (approximately 4 cm length, 0.1 g weight) were collected from each subject and cut as close to the scalp as possible. Hair samples were sent to the La Belle Vie research laboratory and analyzed by established methods, as described previously^17^. For more details, see Supplemental Methods.

The logarithms of mineral levels in the hair were analyzed for all the measures used because logarithms of hair zinc levels were closer to the normal distribution and could alleviate the effects of outliers. For statistical analysis, zinc levels were converted to logarithms that could be used in the analyses, as reported in previous studies, including those from researchers affiliated with institutions in which mineral levels of our hair samples were measured (Research Laboratory, La Belle Vie Inc.)^9,17,36–40^.

### Psychological measures

Following neuropsychological testing, several questionnaires were administered. These tests were chosen because low body zinc levels are associated with attention deficits, autism, and sleep disturbance as described in “Introduction”. The test descriptions in this subsection were largely reproduced from our previous studies^41,42^.

[A] RAPM^43^ is a non-verbal reasoning task and a representative measure of general intelligence. For more details, see our previous study^44^. [B] Systemizing quotient (SQ) and empathizing quotient (EQ). Japanese versions^45^ of the SQ and EQ^46,47^ were administered. EQ score was used as an index of empathizing and SQ score was used as an index of systemizing. [C] The Reverse Stroop and Stroop interference rates of the Stroop task (Hakoda’s version)^48^ was used to measure response inhibition and impulsivity. Hakoda’s version is a matching-type Stroop task requiring subjects to check whether their chosen answers are correct, unlike the traditional oral naming Stroop task. The test consists of two control tasks (word-color and color-word tasks), a Stroop task, and a reverse Stroop task. The reverse Stroop and Stroop interference rates are calculated from these tests. See our previous study for details^49^. [D] S-A creativity test. Creativity as divergent thinking was measured using the S-A creativity test^50^. [E] A computerized digit span task was used to assess working memory for details, see^34^. [F] Sleep disturbance subscale of the *General Heath Questionnaire 30*^51^. [G] The External-Preoccupation Scale^52^, which was used to measure maintenance of external focus on a specific object.

### fMRI task

fMRI was used to map brain activity during cognitive tasks. The descriptions of this task were mostly reproduced from a previous study using the same methods^53^. The n-back task is a typical fMRI task with conditions of 0-back (simple cognitive process) and 2-back (working memory). Subjects were instructed to judge if a stimulus (one of four Japanese vowels presented visually) appearing “n” positions earlier was the same as the current stimulus by pushing a button. In the 0-back task, subjects were instructed to determine whether a presented letter was the same as the target stimulus by pushing a button. We used a simple block design. For more details, see Supplemental Methods.

The effects of inverted-U shaped activity patterns that are observed between the n-back task’s load and activity patterns (namely, the activity increases as the load increases, but when the tasks are too difficult, the activity drops)^54^ are not matter of concerns in this study, as in this study, 2-back tasks are apparently easy enough for all participants and the average accuracy rates are almost about 100%.

### Image acquisition

The MRI acquisition methods were described in our previous study and reproduced below^55^. All MRI data acquisition was performed using a 3 T Philips Achieva scanner.

Diffusion-weighted data were acquired using a spin-echo EPI sequence (TR = 10,293 ms, TE = 55 ms, FOV = 22.4 cm, 2 × 2 × 2 mm^3^ voxels, 60 slices, SENSE reduction factor = 2, number of acquisitions = 1). The diffusion weighting was isotropically distributed along 32 directions (*b* value = 1,000 s/mm^*2*^). In addition, three images with no diffusion weighting (*b* value = 0 s/mm^2^) (b = 0 images) were acquired using a spin-echo EPI sequence (TR = 10,293 ms, TE = 55 ms, FOV = 22.4 cm, 2 × 2 × 2 mm^3^ voxels, 60 slices). FA and MD maps were calculated from the collected images using a commercially available diffusion tensor analysis package on the MR console. For more details, see Supplemental Methods. Descriptions in this subsection were mostly reproduced from a previous study using similar methods^56^.

Forty-two transaxial gradient-echo images (TR = 2.5 s, TE = 30 ms, flip angle = 90°, slice thickness = 3 mm, FOV = 192 mm, matrix = 64 × 64) covering the entire brain were acquired using an echo planar sequence. For the n-back sessions, 174 functional volumes were obtained.

Thorough instructions and thorough fixation by the pad were given as much as possible to prevent head motion during the scan. We did not exclude any subject from the fMRI analyses based on excessive motion during the scan. The subjects were young adults and the scan did not last for long. Only six subjects’ maximum movement from the original point in one of the directions exceeded 3 mm, and removing these subjects from analyses did not substantially alter the significant results of the present study. Furthermore, frame-wise displacement during fMRI scan did not significantly correlate with hair zinc levels after all the other covariates of the whole brain analyses of n-back tasks were controlled (partial correlation analysis, partial correlation coefficient = 0.006, p = 0.854).

### Preprocessing of structural data

Preprocessing and analysis of diffusion and functional activation data were performed using SPM8 implemented in MATLAB. The following descriptions were mostly reproduced from our previous study using similar methods^53^. The methods are summarized below; full details and methodological considerations are provided in the Supplemental Methods. Before analysis, blood-oxygen-level dependent (BOLD) images were realigned and resliced to the mean of the BOLD images, which was then realigned to the mean b = 0 image as previously described ^34^. Because the mean b = 0 image was aligned with the FA image and MD map, the BOLD image, b = 0 image, FA image, and MD map were all aligned. Next, using a previously validated two-step segmentation algorithm of diffusion images and diffeomorphic anatomical registration through an exponentiated lie algebra (DARTEL)-based registration process that utilizes FA signal distribution for normalization^57^, all images—including gray matter segments [regional gray matter density (rGMD) map], white matter segments [regional white matter density (rWMD) map], and cerebrospinal fluid (CSF) segments [regional CSF density (rCSFD) map] of the diffusion images were normalized. The voxel size of the normalized FA images and segmented images was 1.5 × 1.5 × 1.5 mm^3^. The voxel size of the normalized BOLD images was 3 × 3 × 3 mm^3^.

Next, from the average images of the normalized WM segmentation images from 63 subjects, we created a mask image consisting of voxels with a WM signal intensity > 0.99. We then applied this mask image to the normalized FA images, thereby retaining only areas highly likely to be white matter from the normalized FA images. These images were smoothed (6 mm full-width half-maximum) and carried through to the second-level analyses of FA.

### First-level analysis of functional imaging data

The following descriptions were mostly reproduced from our previous study using similar methods^53^. Individual-level statistical analyses were performed using a general linear model. A design matrix was fitted to each participant with one regressor in each task condition (0- or 2-back in the n-back task) using the standard hemodynamic response function. The cue phases of the n-back task were modeled in the same manner, but were not analyzed further. Six parameters obtained by rigid body corrections for head motion were regressed out by adding these variances to the regressor. The design matrix weighted each raw image according to its overall variability to reduce the impact of movement artifacts^58^. We removed low-frequency fluctuations using a high-pass filter with a cut-off value of 128 s. After estimation, beta images of contrasts of 2-back > rest and 0-back > rest were smoothed (8 mm full-width half-maximum) and taken to the second-level analyses.

### Statistical analyses of non-whole-brain analyses

Behavioral data were analyzed using SPSS 22.0 (SPSS Inc., Chicago, IL). The associations of hair zinc levels with psychological outcome measures were tested using multiple regression analyses. The dependent variables were the 16 cognitive variables presented in Table 2. The independent variables comprised sex, age, self-reported height, and BMI (calculated from self-reported height and weight), and hair zinc levels. Results with a threshold of *p* < 0.05, corrected for false discovery rate (FDR) using the graphically sharpened method^59^, were considered statistically significant. Self-reported height, and BMI are included as covariates to exclude confounding effects of these as these variables often correlate with body mineral levels (in the case of zinc, after correcting the effects of age and sex, BMI was significantly negatively correlated with hair zinc levels, p = 0.002, although, height was not). And this is in accordance with our studies^17,60^.

### Whole-brain statistical analysis

We investigated if the imaging measures were associated with individual differences in hair zinc. Whole-brain multiple regression analyses were performed using SPM8.

In the FA analysis, the covariates were sex, age, self-reported height, self-ported weight, BMI, and Zn levels in hair. Analyses were performed within the white matter mask created above.

In the fMRI analyses, the maps of dependent variables were beta estimate images of 2-back > rest contrast and 0-back contrast. Covariates included those used in the FA analysis as well as accuracies and reaction times in the 0-back and 2-back tasks and volume-level mean frame-wise displacement during the scan for the n-back task^61^.

Correction for multiple comparisons was performed using threshold-free cluster enhancement (TFCE)^62^ with randomized (5,000 permutations) nonparametric testing using the TFCE toolbox (https://dbm.neuro.uni-jena.de/tfce/). The family-wise error (FWE) threshold was corrected at *p* < 0.05.

### Ethical statement

All procedures performed in studies involving human participants were in accordance with the ethical standards of the institutional and/or national research committee and with the 1964 Helsinki declaration and its later amendments or comparable ethical standards.

### Ethical approval

This study was approved by the Ethics Committee of Tohoku University.

### Informed consent

Informed consent was obtained from all individual participants included in the study.

## Results

### Basic data

**Figure 1:**
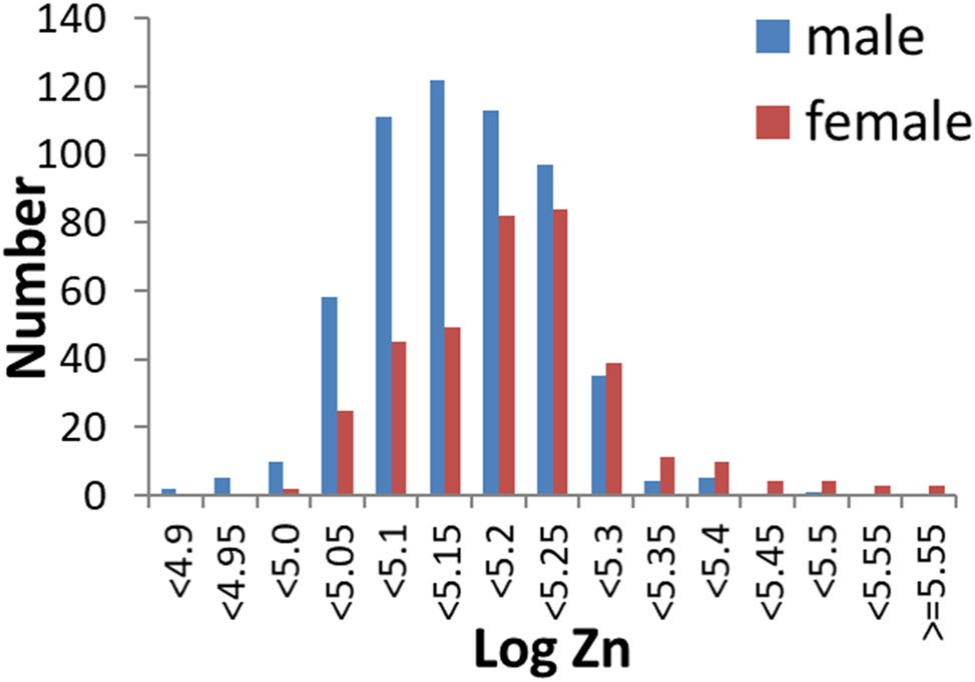
Histograms showing the logarithms of zinc levels in hair for male and female subjects.

The mean and standard deviation for age, general intelligence, and logarithm of hair zinc are presented in Table 1. The distribution of the logarithms of zinc levels in the hair of men and women are presented in Fig. 1.

**Table 1:** Demographics of study participants and statistical values of sex differences.

Measure	Male (N = 563)			Female (N = 361)			Sex differences (independent t test)	
	N	Mean	SD	N	Mean	SD	t value	p value
Age	563	20.83	1.93	563	20.54	1.64	2.301	0.022
RAPM	563	28.77	3.89	563	28.00	3.87	2.933	0.003
Log-Zinc (ppm)	563	5.1401	0.0820	563	5.1900	0.1085	− 7.931	6.28*10^–15^
Empathizing	562	29.89	9.70	361	33.88	10.15	− 5.983	3.14*10^–9^
Systemizing	562	28.28	8.57	361	21.55	7.43	12.24	4.95*10^–32^
Reverse Stroop interference	561	15.65	9.95	361	14.48	9.39	1.781	0.075
Stroop interference	560	7.15	8.92	361	5.83	8.82	2.198	0.028
S-A creativity test	563	36.97	10.69	361	38.67	10.21	− 2.402	0.017
Digit span	560	36.91	7.24	359	34.5	6.56	5.115	3.83^–7^
GHQ30d—sleep disturbance	560	1.23	1.16	361	1.17	1.18	0.779	0.061
Sleep length^a^	562	6.88	1.11	361	6.57	1.06	4.213	2.8^–5^
External-preoccupation	562	27.29	5.24	361	26.67	5.56	1.714	0.087
2-back reaction time	555	6,609.82	1726.68	356	6,860.69	1,820.1	− 2.092	0.037

^a^For the calculation of sleep length, see our previous study^78^.

### Correlations between hair zinc levels and cognitive differences

After correcting for confounding variables and multiple comparisons, zinc levels in hair were not significantly correlated with any of the psychological variables. However, hair zinc showed a trend (P < 0.1, corrected) toward negative correlation with SQ score, the sleep disturbance subscore of the WHOGHQ30, and reverse Stroop interference. The results of all statistical analyses are presented in Table 2.

**Table 2:** Statistical results (beta value, *t*-value, uncorrected *p*-value, *p*-value with FDR correction) for the multiple regression analyses using psychological variables and zinc levels after correction for confounding variables.

Dependent variable	N	Β	*t*	Zinc level	
				*p *(uncorrected)	*p *(FDR)
RAPM^a^	924	− 0.032	− 0.926	0.355	0.355
Empathizing	923	− 0.049	− 1.480	0.139	0.192
Systemizing	923	− 0.074	− 2.324	0.020	0.099
Reverse Stroop interference	922	− 0.071	− 2.090	0.037	0.099
Stroop interference	921	− 0.033	− 0.975	0.330	0.355
S-A creativity test	924	0.007	0.219	0.827	0.633*
Digit span	919	− 0.050	− 1.461	0.144	0.192
GHQ30^b^—sleep disturbance	921	− 0.077	− 2.242	0.025	0.099
Sleep length	923	− 0.057	− 1.670	0.095	0.190
External-preoccupation	923	− 0.015	− 0.430	0.667	0.593*
2-back reaction time	911	0.006	0.163	0.871	0.633*

*FDR* false discovery rate.

*Note that some uncorrected *p*-values are greater than the *p*-values corrected for FDR. These values are indeed correct. In some FDR methods, including the one used in this study, this phenomenon (corrected statistical values exceed the original *p*-values) can occur when some *p*-values among the group of analyzed *p*-values are very strong for the introduction of this phenomena, see^79^.

^a^Raven’s advanced progressive matrices (a general intelligence task).

^b^General Health Questionnaire 30.

### Correlations between hair zinc and FA

**Figure 2:**
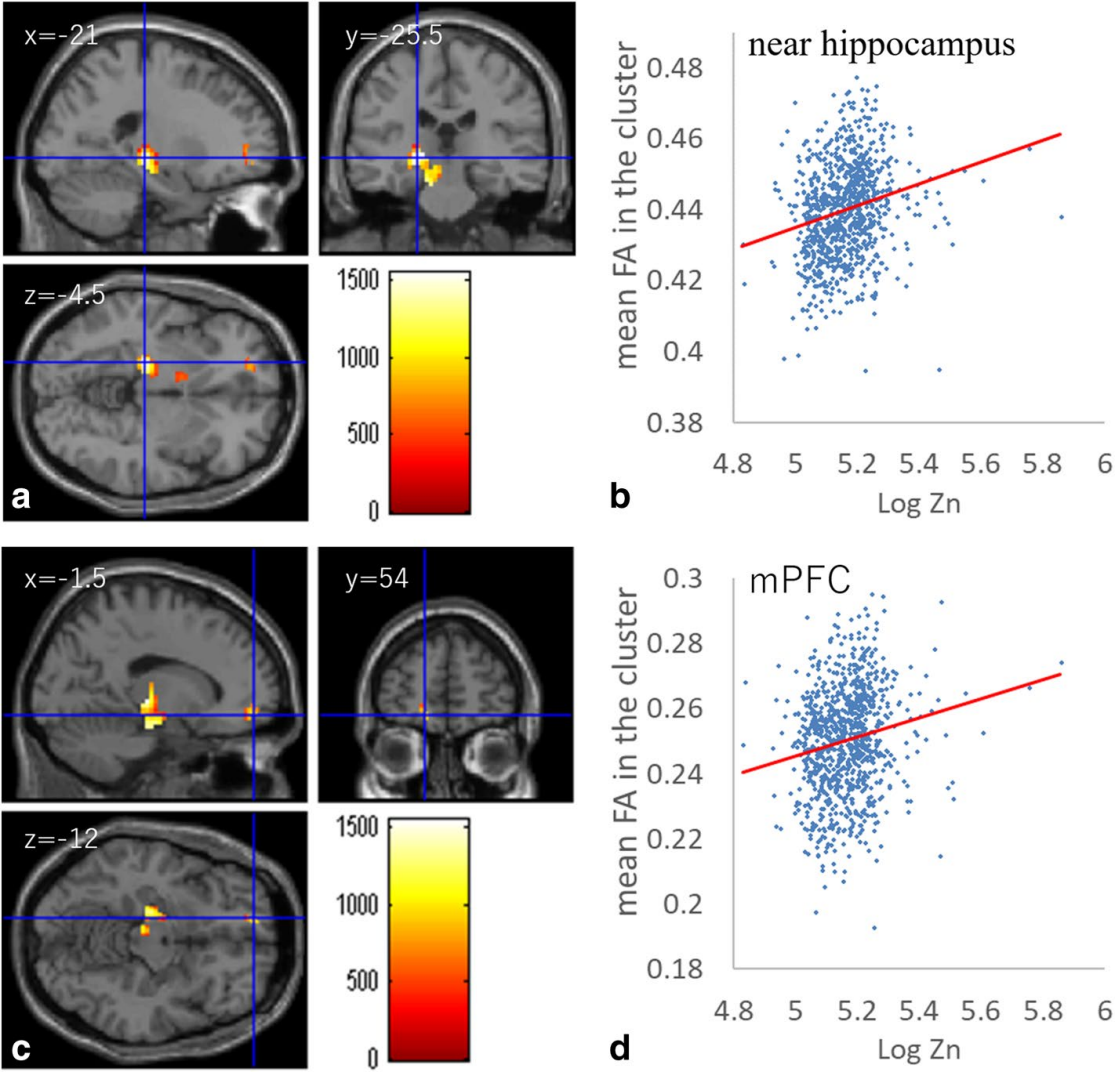
Positive FA correlates with zinc levels in hair. **(a, c)** Regions with significant positive correlations between FA and hair zinc levels are overlaid on a “single subject” T1 image from SPM8. Results were obtained using a threshold of threshold-free cluster enhancement (TFCE) of *p* < 0.05 based on 5,000 permutations. Results were corrected at the whole-brain level. Significant correlations were found in **(a)** white matter areas close to the left hippocampus and **(c)** the area of the medial prefrontal cortex. **(b, d)** Scatter plots of the associations between hair zinc levels and mean FA in the clusters of **(a)** and **(c)**.

Whole-brain multiple regression analysis showed that zinc levels in hair were significantly and positively correlated with FA in white matter areas, including the left fornix, left internal capsule, left corticospinal tract, left cerebellar peduncle, and the white matter area in the left medical prefrontal cortex (mPFC) (Fig. 2a–d, Table 3).

**Table 3:** Brain regions exhibiting significant positive correlations between hair zinc level and FA.

Included large bundles* (number of significant voxels in left and right sides of each anatomical area)	x	y	z	TFCE value	r**	Corrected *p*-value (FWE)	Cluster size (mm^3^)
Corticospinal tract (L:28)/superior cerebellar peduncle (L:1)/Cerebral peduncle (L:451)/posterior limb of internal capsule (L:88)/retrolenticular part of the internal capsule (L:18)/Fornix (cres) (L:33)	− 21	− 25.5	− 4.5	1549.73	0.226	0.0002	4,488.75
NA	− 15	54	− 12	958.62	0.174	0.0002	997.5
Anterior limb of the internal capsule (L:41)	− 9	1.5	− 4.5	590.29	0.061	0.0074	495
NA	− 6	− 9	− 3	422.12	0.06	0.0394	11.25
NA	− 15	− 18	13.5	403.5	0.067	0.048	3.75

*Anatomical labels and significant clusters of major white matter fibers were determined using the ICBM DTI-81 Atlas (https://www.loni.ucla.edu/).

**Simple correlation coefficients of the relationships between hair zinc level and mean FA of the significant clusters. Note that the correlation coefficients of significant areas in whole-brain multiple regression analyses generally do not reflect true effect sizes due to overfitting effects, which are affected by multiple factors including sample size^80^.

### Correlations between hair zinc and brain activity

**Figure 3:**
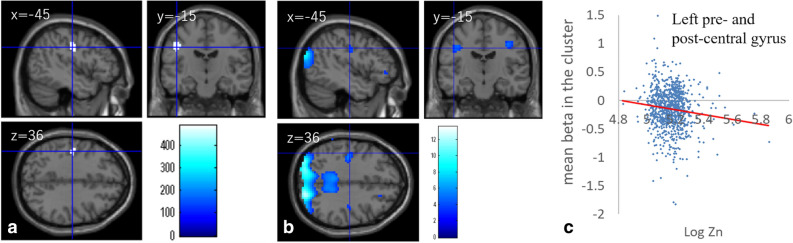
Brain activity correlates with zinc levels in hair. Regions with significant correlations between the brain activity during the 0-back task and hair zinc levels are overlaid on a “single subject” T1 image from SPM8. Results were obtained using a threshold of threshold-free cluster enhancement (TFCE) of *p* < 0.05 based on 5,000 permutations. **(a)** Significant negative correlations were found in the area of the left precentral and postcentral gyrus. **(b)** Regions of deactivation during the 0-back task obtained from the 63 subjects from which the template of the diffusion image was created^57^. Results are overlaid on a “single subject” T1 image from SPM8. Results were thresholded at *p* < 0.001, uncorrected for visualization purposes. **(c)** Scatterplots of the associations between hair zinc levels and mean beta estimates in the clusters of **(a)**.

Whole-brain multiple regression analysis showed that zinc levels in hair were significantly and negatively correlated with brain activity during the 0-back task in an area spreading across the left postcentral gyrus and the left precentral gyrus, which was deactivated during the 0-back task (Fig. 3a–c, Table 4).

**Table 4:** Brain regions exhibiting significant correlations between hair zinc level and brain activity.

Included gray matter areas^a^ (number of significant voxels in the left and right sides of each anatomical area)	x	y	z	TFCE value	r	Corrected *p*-value (FWE)	Cluster size (mm^3^)	Activated areas, deactivated areas during the 2-back task^b^
**Negative correlation between hair zinc level and activity during the 0-back task**								
Postcentral gyrus (L:39)/precentral gyrus (L:5)	− 45	− 15	16	488.61	− 0.119	0.0424	1,080	0%, 97.5%
**Positive correlation between hair zinc level and activity during the 2-back task**								
Posterior cingulum (L:18)/hippocampus (L:1)/precuneus (L:1)	− 12	− 42	15	549.45	0.145	0.0236	1,215	31.11%, 15.56%
Posterior cingulum (R:3)	12	− 42	12	452.33	0.135	0.0472	81	33.33%, 33.33%

^a^Labeling of the anatomical regions of gray matter was based on the WFU PickAtlas Tool (https://www.fmri.wfubmc.edu/cms/software#PickAtlas/)^81,82^ and the PickAtlas automated anatomical labeling atlas option^83^. Temporal pole areas and some other areas include all subregions in the areas of this atlas.

^b^Percentage of voxels activated or deactivated during the 2-back task among the sample of 63 subjects from which the template of the diffusion image was created^57^.

**Figure 4:**
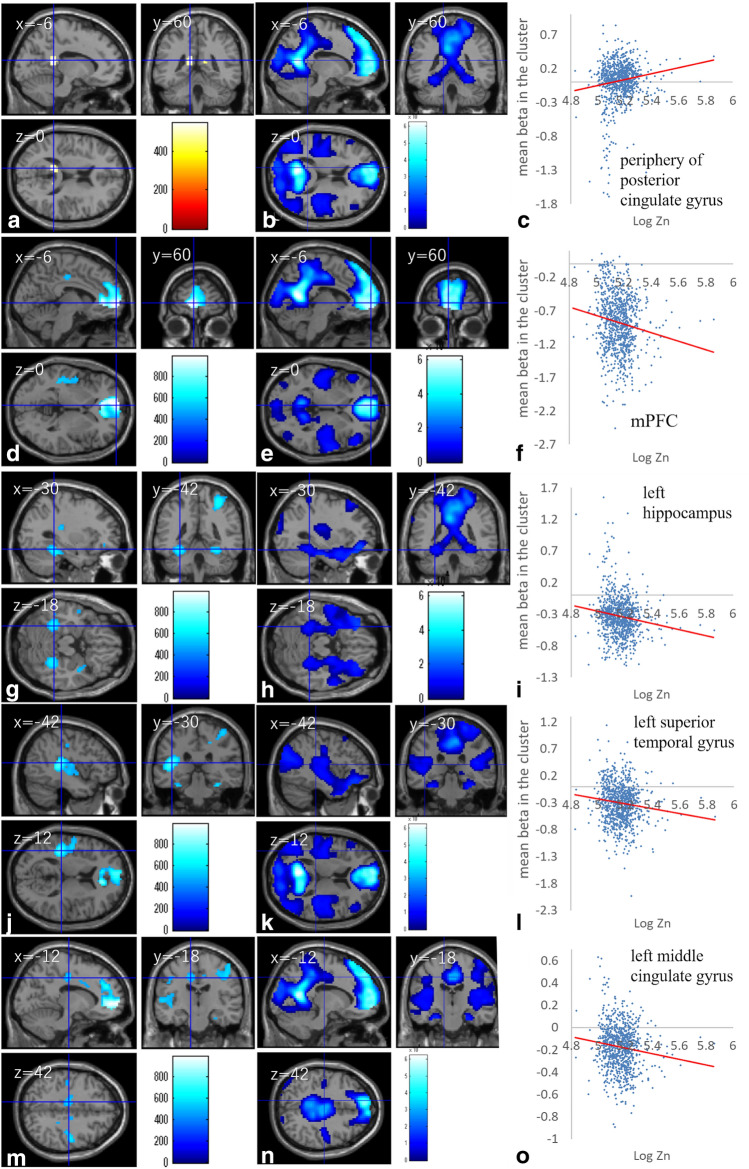
Brain activity correlates with zinc levels in hair. **(a, d, g, j, m)** Regions with significant correlations between the brain activity during the 2-back task and hair zinc levels are overlaid on a “single subject” T1 image from SPM8. Results were obtained using a threshold of threshold-free cluster enhancement (TFCE) of *p* < 0.05 based on 5,000 permutations. Significant positive correlations were observed in **(a)** the peripheral areas of the bilateral posterior cingulate gyrus and significant negative correlations were found in **(d)** the mPFC, **(g)** the left fusiform gyrus, **(j)** left superior temporal gyrus and contingent regions, and **(m)** the left middle cingulate gyrus. **(b, e, h, k, n)** Regions deactivated during the 2-back task obtained from the 63 subjects from which the template of diffusion image was created^57^. Results are overlaid on a “single subject” T1 image from SPM8. Results were obtained using a threshold of threshold-free cluster enhancement (TFCE) of *p* < 0.05 based on 5,000 permutations. **(c, f, i, l, o)** Scatterplots of the associations between hair zinc levels and mean beta estimates in the clusters of **(a), (d), (g), (j)**, and **(m)**.

In addition, zinc levels in hair were significantly and positively correlated with brain activity during the 2-back task in peripheral areas of the posterior cingulate gyrus, which are border areas between the areas deactivated during the 2-back task and areas activated during the 2-back task (Fig. 4a–c, Table 4).

Whole-brain multiple regression analysis also showed that zinc levels in hair were significantly and negatively correlated with brain activity during the 2-back task in the extensive areas deactivated during the 2-back task. The areas of significant correlation included the cluster around the mPFC, the cluster around the left superior temporal gyrus, left Rolandic operculum, left Heschl gyrus, left insula, left precentral and postcentral gyrus, the cluster mainly around the right precentral gyrus, postcentral gyrus, and right superior parietal lobule, bilateral clusters mainly around the bilateral fusiform gyrus, bilateral clusters in the bilateral middle cingulate gyrus, the cluster in the right insula, and the cluster in the left orbitofrontal gyrus (Fig. 4d–o, Table 5). We used permutation based corrections for multiple comparisons and effects of existence of outliers are taken care of in corrections of multiple comparisons.

**Table 5:** Brain regions exhibiting significant negative correlation between hair zinc level and brain activity during the 2-back task.

Included gray matter areas (number of significant voxels in the left and right sides of each anatomical area)	x	y	z	TFCE value	r	Corrected *p*-value (FWE)	Cluster size (mm^3^)	Activated areas Deactivated areas
Anterior cingulum (L:167, R:110)/middle frontal medial area (L:48, R:49)/superior frontal medial area (L:312, R:133)/superior frontal orbital area (L:3)/superior frontal other areas (L:27, R:1)	− 6	60	0	984.85	− 0.147	0.0022	22,950	0%, 99.76%
Heschl gyrus (L:59)/insula (L:43)/postcentral gyrus (L:83)/precentral gyrus (L:28)/Rolandic operculum (L:105)/supramarginal gyrus (L:2)/middle temporal gyrus (L:26)/superior temporal gyrus (L:224)/	− 42	− 30	12	745.61	− 0.124	0.0074	14,958	0%, 91.7%
Middle cingulum (R:3)/middle frontal other areas (R:5)/inferior parietal lobule (R:17)/superior parietal lobule (R:29)/postcentral gyrus (R:236)/precentral gyrus (R:160)	33	− 42	57	653.39	− 0.117	0.0128	12,420	3.26%, 83.91%
Fusiform gyrus (L:116)/parahippocampal gyrus (L:1)/inferior temporal gyrus (L:6)/cerebellum (L:25)	− 30	− 42	− 18	617.27	− 0.147	0.018	3,753	0%, 87.77%
Fusiform gyrus (R:125)/hippocampus (R:2)/parahippocampal gyrus (R:16)/cerebellum (R:29)	27	− 45	− 18	599.87	− 0.119	0.0202	4,347	0.62%, 90.06%
Middle cingulum (L:43)/supplemental motor area (L:1)	− 12	− 18	42	540.53	− 0.125	0.0282	1728	1.56%, 84.38%
Insula (R:21)	39	0	− 15	510.26	− 0.107	0.0328	1,188	0%, 100%
Inferior frontal orbital area (L:7)/middle frontal orbital area (L:6)	− 33	39	− 12	477.06	− 0.108	0.0416	297	0%, 100%
Middle cingulum (L:4)	− 12	12	30	467.71	− 0.134	0.0444	594	18.18%, 4.55%
Middle cingulum (R:10)	12	− 15	45	462.49	− 0.089	0.046	270	0%, 100%
Middle temporal gyrus (L:9)	− 54	0	− 21	460.45	− 0.114	0.0476	33.75	0%, 100%
Superior frontal other areas (L:2)	− 15	39	51	453.88	− 0.108	0.0496	54	0%, 100%
Inferior temporal gyrus (L:1)	− 48	− 39	− 18	453.14	− 0.102	0.05	27	0%, 100%

In this study, our focus was TID in the DMN, and TID in the DMN occurs regardless of the whether the task is 2-back or 0-back in mostly similar areas, although the magnitude is different. Furthermore, differences in the brain activity between patients with schizophrenia and control subjects were mostly similarly regardless of whether the task was 0-back task or 2-back task, including in the areas of DMN (i.e., subtracting the activity during the 0-back task from the brain activity during the 2-back task substantially eliminates the group differences)^33,63^. Therefore, we did not primarily analyze the contrast of (2-back – 0-back) as in case of another study that focused on TID in the DMN^33^. However, additional analysis involving this contrast revealed that after correcting the same covariates of the whole brain analyses of 2-back and 0-back, the brain activity of the contrast of (2-back – 0-back) showed a significant negative correlation in the area of mPFC [x, y, z =  − 12, 63, 3, TFCE score = 691.14, P = 0.012, corrected for FWE (permutation using TFCE), 6,939 mm^3^] and a significant positive correlation in the area spreading around the posterior cingulate gyrus and the left hippocampus [x, y, z =  − 12, − 42, 15, TFCE score = 667.14, P = 0.013, corrected for FWE (permutation using TFCE), 2,970 mm^3^]. The results indicated that the significant results of the whole brain analyses of the brain activity of the contrast of (2-back – 0-back) are part of the significant results of the correlation between 2-back and hair zinc level (i.e., Fig. 4).

## Discussion

The present study revealed brain activity during cognitive tasks and white matter structural properties in a large cohort of young adults in a developed country. Partly consistent with our hypothesis, greater hair zinc level was associated with lower brain activity during a working memory task in extensive areas in the DMN. Since these areas are deactivated during the task, the results suggest that greater hair zinc is associated with greater TID in the DMN. Although negative correlations were found in many areas of the DMN, there were few significant correlations in the hippocampus. Greater hair zinc was also associated with lower brain activity in the left postcentral gyrus and the left precentral gyrus, which was deactivated during the simple cognitive task (greater TID in this area). Further, partly consistent with our hypothesis, greater hair zinc was positively correlated with FA in white matter areas, mainly the left fornix, left internal capsule, left corticospinal tract, left cerebellar peduncle, and white matter area in the left mPFC. Finally, partly consistent with our hypothesis, greater hair zinc was tended to be associated with lower systemizing which is one of the key traits of autistic spectrum disorder, lower sleep disturbance, and lower reverse Stroop interference scores.

Previous work has reported that greater hair zinc is associated with greater TID^64^ in extensive areas of the DMN. Although we can only speculate on the micro-level mechanisms from our macro-level MRI observations, the results are congruent with the idea that zinc inhibits both excitatory and inhibitory receptors, that the dominant role of zinc in the normal brain is to reduce excitability^3,4^, and that TID in the DMN is associated with brain excitability/inhibition underlain by glutamate and GABA^32^. It is possible that greater zinc availability affects glutamate and GABA receptors and reduces excitability/inhibition, resulting in enhancement of the TID in the DMN. In addition,proteins of the ProSAP/Shank family act as major organizing scaffolding elements within the postsynaptic density of excitatory synapses^65^, and mutations in the genes of these proteins are associated with autism and Phelan-McDermid syndrome, which is characterized by the occurrence of seizures and autism-like symptoms for summary, see ref^66^. Zinc ion, which is released from the presynaptic vesicles and influxed into the postsynaptic compartment^3^, interplays with proteins of the ProSAP/Shank family and also plays a key role in their functions^12^. An animal model study has shown that acute and prenatal zinc deficiencies lead to the loss of proteins of the ProSAP/Shank family and autism spectrum disorder-related symptoms^66^. The observed associations are congruent with findings of low hair zinc in autistic children and schizophrenia patients^9^ and the notion that an increased excitability/inhibition ratio plays a key role in the pathology of these conditions^7,8^. Further, zinc level was associated with lower systemizing in the present study, which is a characteristic of autistic spectrum disorders. Thus, our psychological finding may be congruent with previous clinical findings. Finally, greater zinc levels were associated with reduced sleep disturbance in the present study, consistent with previous findings that zinc is important for sleep quality, possibly due to its ability to suppress brain excitability^6^. Interestingly, primary insomnia is also characterized by reduced TID in the DMN^67^ and better Stroop performance is associated with greater GABA levels in the brain^68^. Therefore, hair zinc’s association with low characteristics of autism and sleep disturbance may be parallel to the reduced TID in the DMN and underlain by reduced neural excitability. These speculations may be congruent with the suggestion that zinc deficiency leads to autism-like symptoms, and zinc supplementation may help in improving this problem through animal model studies^66,69^, through animal model studies^66,69^, a large sample study of reduced hair zinc levels in children with autism spectrum disorders^9^. However, the results revealed by intervention studies of zinc are mixed^69^ and future studies should confirm these notions.

Although extensive areas in several brain regions deactivated during the working memory task showed negative correlations between fMRI activity and hair zinc, such associations were mostly lacking in the hippocampus, which also showed deactivation during the working memory task. As described above, the hippocampus was a region of particular interest in this study. Although it is difficult to make inferences from negative findings in whole-brain imaging analyses using stringent thresholds, we can speculate about the lack of results for the hippocampus. Increased availability of zinc largely works to reduce excitability in the brain; however, zinc deprivation produces deficits in hippocampal activation and abrogates LTP, which are important activities for memory formation (and may also be important for working memory)^15^. Therefore, the role of zinc in deactivation during the working memory task may have distinct characteristics. Future studies are needed to investigate the effects of hair zinc on brain activity during a task that recruits the hippocampus more so than during rest.

Partly consistent with our hypothesis, greater hair zinc was associated with greater FA in the white matter area of the left fornix, left internal capsule, left corticospinal tract, left cerebellar peduncle, and white matter area in the left medical prefrontal cortex. This is congruent with previous studies suggesting the importance of zinc in myelination^35^, as increased myelination is thought to lead to greater FA in DTI^70^. In addition, it has been well established that myelination continues even in adulthood^71–73^. Moreover, human studies involving DTI have shown experience-dependent white matter plasticity measured via FA, even in adulthood^74,75^. Although the importance of zinc in myelination has been discussed in many reviews^4,76^, its exact mechanism remains unclear. Thus, we can only speculate. Among the regions showing significant correlations, the fornix acts as a major output tract of the hippocampus. In mice, induction of myelination by neural activity has been demonstrated both in vivo and in vitro (Deremens et al., 1996). As stated earlier, zinc is important for facilitation of activity critical to memory formation in the hippocampus^1,14,15^. Thus, one intriguing possibility is that facilitated activity involving the hippocampus enhances myelination and leads to FA in relevant areas, such as the fornix. Consistently, partial correlation analysis revealed that after controlling for hair zinc levels and all covariates of whole brain analyses of n-back tasks and the FA analysis, mean beta estimate of the significant positive cluster of the area close to the hippocampus/posterior cingulate gyrus (Fig. 4a) and the mean FA value of the significant cluster of the area close to the left hippocampus (Fig. 2a) were significantly and positively correlated (partial correlation coefficient = 0.151, P < 0.001). However, this is pure speculation. Other studies have shown that zinc binds to CNS myelin basic protein in the presence of phosphate, which results in aggregation of this protein and may affect myelination^77^. Ultimately, we cannot determine micro-level mechanisms from macro-level neuroimaging data, and so future studies are needed to investigate such issues. As such, the effects of zinc on white matter microstructural properties are assumed to be independent from the effects of zinc on greater deactivation, and this is not particularly strange given the diverse mechanisms each nutrient affects.

There is at least one limitation of this study. As it is a cross-sectional macro-level neuroimaging study, we cannot determine causal relationships or the underlying micro-level neural mechanisms behind the observed associations involving hair zinc levels. Future studies using other methods, such as interventions involving zinc supplementation or animal experiments, could complement the present findings.

In conclusion, we investigated the associations of zinc levels in hair with cognitive domains, brain activity, and FA in a relatively large cohort of young adults. Greater zinc levels were associated with lower TID in the DMN and greater FA in white matter areas near the hippocampus and areas like the posterior limbs of the internal capsule. Greater hair zinc showed a tendency of association with lower systemizing which is a key characteristic of autistic spectrum disorders, lower sleep disturbance, and lower reverse Stroop interference. These findings advance previous non-neuroimaging findings of zinc’s associations with excitability, disorders associated with excitability, and myelination.

## Supplementary information

Supplementary Information.

## Data Availability

All the experimental data obtained in the experiment of this study will be available for the studies that were admitted in the ethics committee of Tohoku University’s medical faculty. All the data sharing should be first admitted by the ethics committee of Tohoku University’s medical faculty.
