# Supplementary material for: Succeeding in deactivating: associations of hair zinc levels with functional and structural neural mechanisms
Source: Sci Rep. 2020 Jul 23;10:12364. doi: 10.1038/s41598-020-69277-4 (PMC7378227; doi:10.1038/s41598-020-69277-4)
Supplement: Supplementary file 1 — Supplementary Information. [file 41598_2020_69277_MOESM1_ESM.pdf]

Succeeding in deactivating: associations of hair zinc levels with functional and structural neural mechanisms

Hikaru Takeuchi[a], Yasuyuki Taki[a,b,c], Rui Nouchi[d,e,f], Ryoichi Yokoyama[g], Yuka Kotozaki[h], Seishu Nakagawa[i,j], Atsushi Sekiguchi[b,k], Kunio Iizuka[l], Sugiko Hanawa[i], Tsuyoshi Araki[m], Carlos Makoto Miyauchi[n], Kohei Sakaki[f], Takayuki Nozawa[o], Shigeyuki Ikeda[p], Susumu Yokota[a], Magistro Daniele[q], Yuko Sassa[a], Ryuta Kawashima[a,f,i]

[a] *Division of Developmental Cognitive Neuroscience, Institute of Development, Aging and Cancer, Tohoku University, Sendai, Japan*

[b] *Division of Medical Neuroimaging Analysis, Department of Community Medical Supports, Tohoku Medical Megabank Organization, Tohoku University, Sendai, Japan*

[c] *Department of Radiology and Nuclear Medicine, Institute of Development, Aging and Cancer, Tohoku University, Sendai, Japan*

[d] *Creative Interdisciplinary Research Division, Frontier Research Institute for Interdisciplinary Science, Tohoku University, Sendai, Japan*

[e] *Human and Social Response Research Division, International Research Institute of Disaster Science, Tohoku University, Sendai, Japan*

[f] *Department of Advanced Brain Science, Institute of Development, Aging and Cancer, Tohoku University, Sendai, Japan*

[g] *School of Medicine, Kobe University, Kobe, Japan*

[h] *Division of Clinical research, Medical-Industry Translational Research Center, Fukushima Medical University School of Medicine, Fukushima, Japan*

[i] *Department of Human Brain Science, Institute of Development, Aging and Cancer, Tohoku University, Sendai, Japan*

[j] *Division of Psychiatry, Tohoku Medical and Pharmaceutical University, Sendai, Japan*

[k] *Department of Behavioral Medicine, National Institute of Mental Health, National*

Center of Neurology and Psychiatry, Tokyo, Japan

[l]*Department of Psychiatry, Tohoku University Graduate School of Medicine, Sendai, Japan*

[m]*ADVANTAGE Risk Management Co., Ltd.*

[n] *Department of Language Sciences, Graduate School of Humanities, Tokyo Metropolitan University, Tokyo, Japan*

[o] *Research Center for the Earth Inclusive Sensing Empathizing with Silent Voices, Tokyo Institute of Technology, Tokyo, Japan*

[p]*Department of Ubiquitous Sensing, Institute of Development, Aging and Cancer, Tohoku University, Sendai, Japan*

[q]*Department of Sport Science, School of Science and Technology, Nottingham Trent University, Nottingham, UK*

Corresponding author (also Lead Contact):

Hikaru Takeuchi

Division of Developmental Cognitive Neuroscience, IDAC, Tohoku University

4-1 Seiryo-cho, Aoba-ku, Sendai 980-8575, Japan

Tel/Fax: +81-22-717-7988

E-mail: takehi@idac.tohoku.ac.jp

Short title: Zinc, deactivation, and white matter property

Keywords: Hair minerals, zinc, diffusion tensor imaging, fractional anisotropy, default mode network, hippocampus

Supplemental online material

Supplemental Methods

Additional subject details. Some subjects who participated in this study also participated in our intervention studies (psychological data and imaging data recorded before the intervention were used in the present study) [1]. Psychological tests and MRI scans not described here were performed together with those described in the present study. All subjects were university students, postgraduates, or university graduates of less than one year's standing. All subjects had normal vision and none had neurological or psychiatric illnesses. Handedness was evaluated using the Edinburgh Handedness Inventory [2].

Subjects were instructed to get sufficient sleep, maintain their condition, eat sufficient breakfast, and consume their usual amount of caffeinated foods/drinks on the day of cognitive testing and MRI scans. Subjects were also instructed to avoid alcohol the night before the assessment.

The descriptions in this subsection were mostly reproduced from one of our other studies from the same project using the same methods [3].

Hair acquisition and hair mineral analysis

Scalp hair samples (approximately 4 cm length, 0.1 g weight) were collected from each subject, with the hair cut as close to the scalp as possible. Hair samples were sent to the La Belle Vie research laboratory and analyzed by established methods [4-9], as described below.

A 75-mg hair sample was weighed in a 50-ml plastic tube and then washed twice with acetone and once with 0.01% Triton solution, as recommended by the Hair Analysis Standardization Board [10]. The washed hair sample was mixed with 10 ml 6.25% tetramethylammonium hydroxide (Tama Chemical) and 50 µl 0.1% gold solution (SPEX Certi Prep.) and dissolved at 75°C with shaking for 2 h. After cooling the solution to room temperature, the internal standard (Sc, Ga, and In) solution was added, and adjusting the volume gravimetrically, the solution was used for mineral analysis. Mineral concentrations were measured by inductively coupled plasma mass spectrometry (Agilent-7500ce) by the internal standard method [4, 11, 12] and are expressed as ng/g hair (ppb). For quality control, we used human hair certified reference materials from the National Institute for Environmental Studies of Japan (NIES CRM no. 13) [13].

The description in this subsection was reproduced from our previous work using the same method [14].

We also obtained information on the last time subjects underwent hair coloring, perming, or bleaching. The possible answers were (a) within 1 month, (b) within 1–2 months, (c) within 2–3 months, and (d) within 3–6 months, and (e) not performed within 6 months. As the hair samples (approximately 4 cm long) were cut as close to the scalp as possible, based on hair growth, the answers were coded as follows: (a) = 3, (b) = 2, (c) = 1, (d) = 0.5, and (e) = 0. Analyses that included history of coloring, perming, and bleaching as covariates did not substantially affect the strength of the significant

associations in the present study. We did not provide any instruction regarding shampoo use nor collected the information on shampoo use. In Japan, popular shampoos like “Merit” (Kao Corporation, Tokyo, Japan) had been using zinc pyrithione long time ago (in the case of Merit, it was used until 2006, long before the project started). But it was no longer used in popular shampoos as far as we know, though still not-popular some medicinal shampoos seem to be using zinc pyrithione, but the use was apparently very rare in the young. And the official questionnaire for this hair sampling does not include items related to shampoo.

Details of the N-back fMRI task. Participants received instructions for the tasks and practiced the tasks before entering the MRI scanner. During scanning, they viewed stimuli on a screen via a mirror mounted on a head coil. Visual stimuli were presented using Presentation Software (Neurobehavioral Systems, Inc., Albany, CA, USA). A fiber-optic light-sensitive key press interface with a button box was used to record participants’ task responses.

Two conditions were used: 0-back and 2-back. Each condition had six blocks, and all N-back tasks were performed in one session. Subjects were instructed to recall visually presented stimuli (four Japanese vowels) presented “n” stimuli before the currently presented stimulus (e.g., participants had to recall the letter presented two letters earlier for the 2-back task or the currently presented letter for the 0-back task). Two buttons were used during the 0-back task: subjects were instructed to push the first button when the defined target stimuli were presented and the second button when non-target stimuli

were presented. During the 2-back task, subjects were instructed to push the first button when the currently presented stimulus and the stimulus presented two stimuli earlier were the same, and to push the second button when the currently presented stimulus and the stimulus presented two stimuli earlier differed. Since the four stimuli were presented randomly, the ratio of matched trials to unmatched trials was 1:3 on average. Our version of the N-back task was designed to require individuals to push buttons continuously during the task period. The task level of the memory load was presented above the stimuli for 2 s before the task started and remained visible and unchanged during the task period (cue phase). Each letter stimulus was presented for 0.5 s with a fixation cross presented for 1.5 s between items. Each block consisted of 10 stimuli. Thus, each block lasted 20 s. A baseline fixation cross was presented for 13 s between the last task item and the presentation of the next task level of the memory load (start of the cue phase). Thus, the rest period lasted for 15 s (13 s + 2 s). There were six blocks for each 2- and 0-back condition. The descriptions in this subsection were mostly reproduced from another study of ours from the same project using the same methods [15].

Details of diffusion image acquisition. The acquisitions for phase correction and signal stabilization were not used as reconstructed images. MD and FA maps were calculated from the collected images using a commercially-available diffusion tensor analysis package on the MR console. This method has been used in many of our previous studies [16-20]. The image-generated results were congruent with those of previous studies using other methods [21, 22], suggesting the validity of this method. The procedures involved

correction for motion and distortion caused by eddy currents. Calculations were performed according to a previously proposed method [23]. Descriptions in this subsection were mostly reproduced from a previous study using similar methods [24].

Preprocessing of imaging data

Preprocessing and analysis of functional activation data were performed using SPM8 implemented in MATLAB. Descriptions in this subsection were mostly reproduced from a previous study using similar methods [15]. Before analysis, BOLD images were realigned and resliced to the mean BOLD image, which was then realigned to the mean of b = 0 images, as described previously [25]. Because the mean b = 0 image was aligned with the FA image and MD map, the BOLD image, b = 0 image, FA image, and MD map were all aligned. Subsequently, using a previously validated two-step segmentation algorithm of diffusion images and diffeomorphic anatomical registration through an exponentiated lie algebra (DARTEL)-based registration process [17], all images—including gray matter segments [regional gray matter density (rGMD) map], white matter segments [regional white matter density (rWMD) map], and cerebrospinal fluid (CSF) segments [regional CSF density (rCSFD) map] of the diffusion images—were normalized.

The details of these procedures, which were also described in our previous study [17], are as follows. Using the new segmentation algorithm implemented in SPM8, FA images of each individual were segmented into six tissues (first new segmentation). The default parameters and tissue probability maps were used in this process, except that affine regularization was performed using the International Consortium for Brain Mapping template for East Asian brains and the sampling distance (approximate distance between sampled points when estimating the model parameters) was 2 mm. We then synthesized the FA image and MD map. In the synthesized image, the area with a WM tissue probability >0.5 in the abovementioned new segmentation process was the FA image multiplied by −1 (hence, the synthesized image shows very clear contrast between WM and other tissues); the remaining area is the MD map (for details of this procedure, see below). The synthesized image from each individual was then segmented using the new segmentation algorithm implemented in SPM8 with the same parameters as above (second new segmentation). This two-step segmentation process was adopted because the FA image has a relatively clear contrast between GM and WM, as well as between WM and CSF, and the first new segmentation step can segment WM from other tissues. On the other hand, the MD map has clear contrast between GM and CSF and the second new segmentation can segment GM. Since the MD map alone lacks

clear contrast between WM and GM, we must use a synthesized image (and the two-step segmentation process).

We then proceeded to the DARTEL registration process implemented in SPM8. We used the DARTEL import image of the GM tissue probability map produced in the second new segmentation process as the GM input for the DARTEL process. The WM input for the DARTEL process was created as follows. First, the raw FA image was multiplied by the WM tissue probability map from the second new segmentation process within the areas with a WM probability >0.5 (signals from other areas were set to 0). Next, the FA image * WM tissue probability map was coregistered and resliced to the DARTEL import WM tissue probability image from the second segmentation. The template for the DARTEL procedures was created using imaging data from 63 subjects who participated in the experiment in our lab [25] and were included in the present study (meaning that they have the same characteristics as the subjects in this study). The first reason why we created the DARTEL template from the images of a subset of all subjects (63 subjects) and not from all subjects is because this is a large sample for creating a template compared to previous studies and thus cannot be considered problematic. The second reason is that the project in which the subjects participated is ongoing, and the DARTEL processes—especially our processes—require vast amounts

of time and the resultant images require large storage resources; thus, we cannot reprocess the images of all subjects and add newer images whenever we change the number of subjects. Next, using this existing template, the DARTEL procedures were performed for all subjects in this study. In these procedures, the parameters were changed as follows to improve accuracy. The number of Gauss–Newton iterations performed within each outer iteration was set to 10 and, in each outer iteration, we used 8-fold more timepoints to solve the partial differential equations than the default values. The number of cycles used by the full multi-grid matrix solver was set to 8. The number of relaxation iterations performed in each multi-grid cycle was also set to 8. The resultant synthesized images were spatially normalized to MNI space. Using these parameters, the raw FA map, rGMD, and rWMD map from the abovementioned second new segmentation process were normalized to give images with $1.5 \times 1.5 \times 1.5$ mm^3 voxels. The FA image * WM tissue probability map was used in the DARTEL procedures because it includes different signal intensities within WM tissues and the normalization procedure can take advantage of intensity differences to adjust the image to the template from the perspective of the outer edge of the tissue and within the WM tissue. No modulation was performed in the normalization procedure.

The voxel size of the normalized FA images, MD images, and segmented images was 1.5 × 1.5 × 1.5 mm^3. The voxel size of the normalized BOLD images was 3 × 3 × 3 mm^3. Normalized rGMD maps were smoothed (3 mm full-width half-maximum) and taken to the second-level analyses of functional activities.

Next, we created average images of normalized rGMD and rWMD images from the normalized rGMD and rWMD images from the subset of the entire sample (63 subjects) [17]. From the average image of normalized WM segmentation images from the 63 subjects mentioned above, we created mask image consisting of voxels with a WM signal intensity > 0.99. We then applied this mask image to the normalized FA image, thereby only retaining areas highly likely to be white matter. These images were smoothed (6 mm full-width half-maximum) and carried through to the second-level analyses of FA. As described previously [17], through application of the mask, images unlikely to be WM or border areas between WM and other tissues were removed. The FA images were not affected by signals from tissues other than WM even after smoothing. This is important considering that, in these areas, WM volume and FA are highly correlated [26] and the FA map supposedly reflects the extent of WM. Further, differences in WMC compared with other tissues among individuals can be ignored after application of this mask because, within the masks, all voxels show very high

white mater probability. For validation of these preprocessing methods and comparison with other methods, see the supplementary online material of our previous study [17].

Through these procedures, we believe that we successfully mitigated or removed the problems of voxel-based analysis of FA analysis raised by Smith et al [27]. These problems include (a) misalignment within white matter tissue (addressed by new segmentation processes and DARTEL processes that utilized difference in signal distribution within white matter using the FA signal) and (b) the effects of different tissue types and partial volume effects (addressed by new segmentation processes, the DARTEL processes, and application of the mask confined to images highly likely to be white matter (in the case of MD maps, white matter or gray matter)). Through these methods, the white matter of DTI images as well as the gray matter areas of DTI images become available for analysis. We avoided co-registration of DTI images to T1-weighted structural images because the shapes differ due to the unignorable distortion of EPI images in 3T MRI.

References

1. Takeuchi H, Taki Y, Nouchi R, Hashizume H, Sekiguchi A, Kotozaki Y *et al.* Effects of Multitasking-Training on Gray Matter Structure and Resting State Neural Mechanisms. *Hum Brain Mapp* 2014; **35**(8)**:** 3646-3660.

2. Oldfield RC. The assessment and analysis of handedness: the Edinburgh inventory. *Neuropsychologia* 1971; **9**(1)**:** 97-113.

3. Takeuchi H, Taki Y, Nouchi R, Sekiguchi A, Hashizume H, Sassa Y *et al.* Degree centrality and fractional amplitude of low-frequency oscillations associated with Stroop interference. *Neuroimage* 2015; **119**(1)**:** 197-209.

4. Yasuda H, Yonashiro T, Yoshida K, Ishii T, Tsutsui T. Mineral Imbalance in Children with Autistic Disorders. *Biomedical research on trace elements* 2005; **16**(4)**:** 285-292.

5. Yasuda H, Yoshida K, Segawa M, Tokuda R, Tsutsui T, Yasuda Y *et al.* Metallomics study using hair mineral analysis and multiple logistic regression analysis: relationship between cancer and minerals. *Environ Health Prevent Med* 2009; **14**(5)**:** 261-266.

6. Yasuda H, Yoshida K, Yasuda Y, Tsutsui T. Infantile zinc deficiency: association with autism spectrum disorders. *Scientific reports* 2011; **1:** 129.

7. Munakata M, Onuma A, Haginoya K, Kobayashi Y, Yokoyama H, Fujiwara I *et al.* Reduced exposure to mercury in patients receiving enteral nutrition. *The Tohoku Journal of Experimental Medicine* 2006; **210**(3)**:** 209-212.

8. Yasuda H, Yoshida K, Yasuda Y, Tsuts T. Two Age-Related Accumulation Profiles of Toxic Metals. *Curr Aging Sci* 2012; **5**(2)**:** 105-111.

9. Yasuda H, Yoshida K, Segawa M, Tokuda R, Yasuda Y, Tsutsui T. High accumulation of aluminum in hairs of infants and children. *Biomedical research on trace elements* 2008; **19**(1)**:** 57-62.

10. Cranton E, Bland J, Chatt A, Krakovitz R, Wright J. Standardization and interpretation of human hair for elemental concentrations. *Journal of Holistic Medicine* 1982; **4**(1)**:** 10-20.

11. Yasuda H, Yonashiro T, Yoshida K, Ishii T, Tsutsui T. High toxic metal levels in scalp hair of infants and children. *Biomed Res Trace Elem* 2005; **16**(1)**:** 39-45.

12. Yasuda H, Yoshida K, Fukuchi K, Tokuda R, Tsutsui T, Yonei Y. Association between aging and minerals in male Japanese adults. *Anti Aging Med* 2007; **4:** 38-42.

13. Yoshinaga J, Morita M, Okamoto K. New human hair certified reference material for methylmercury and trace elements. *Fresenius J Anal Chem* 1997; **357**(3)**:** 279-

283.

14. Takeuchi H, Taki Y, Sekiguchi A, Nouchi R, Kotozaki Y, Nakagawa S *et al.* Association of hair iron levels with creativity and psychological variables related to creativity. *Frontiers in Human Neuroscience* 2013; **7, Article 875:** 1-9.

15. Takeuchi H, Tomita H, Taki Y, Kikuchi Y, Ono C, Yu Z *et al.* Cognitive and neural correlates of the 5-repeat allele of the dopamine D4 receptor gene in a population lacking the 7-repeat allele. *Neuroimage* 2015; **110:** 124-135.

16. Takeuchi H, Taki Y, Sassa Y, Hashizume H, Sekiguchi A, Nagase T *et al.* White matter structures associated with emotional intelligence: Evidence from diffusion tensor imaging. *Hum Brain Mapp* 2013; **34**(5)**:** 1025-1034.

17. Takeuchi H, Taki Y, Thyreau B, Sassa Y, Hashizume H, Sekiguchi A *et al.* White matter structures associated with empathizing and systemizing in young adults. *Neuroimage* 2013; **77**(15)**:** 222-236.

18. Takeuchi H, Sekiguchi A, Taki Y, Yokoyama S, Yomogida Y, Komuro N *et al.* Training of Working Memory Impacts Structural Connectivity. *J Neurosci* 2010; **30**(9)**:** 3297-3303.

19. Takeuchi H, Taki Y, Sassa Y, Hashizume H, Sekiguchi A, Fukushima A *et al.* White matter structures associated with creativity: Evidence from diffusion tensor

imaging. *Neuroimage* 2010; **51**(1)**:** 11-18.

20. Takeuchi H, Taki Y, Sassa Y, Hashizume H, Sekiguchi A, Fukushima A *et al.* Verbal working memory performance correlates with regional white matter structures in the fronto-parietal regions. *Neuropsychologia* 2011; **49**(12)**:** 3466-3473

21. Taki Y, Thyreau B, Hashizume H, Sassa Y, Takeuchi H, Wu K *et al.* Linear and curvilinear correlations of brain white matter volume, fractional anisotropy, and mean diffusivity with age using voxel-based and region of interest analyses in 246 healthy children. *Hum Brain Mapp* 2013; **34**(8)**:** 1842-1856.

22. Barnea-Goraly N, Menon V, Eckert M, Tamm L, Bammer R, Karchemskiy A *et al.* White matter development during childhood and adolescence: a cross-sectional diffusion tensor imaging study. *Cereb Cortex* 2005; **15**(12)**:** 1848-1854.

23. Le Bihan D, Mangin JF, Poupon C, Clark CA, Pappata S, Molko N *et al.* Diffusion tensor imaging: concepts and applications. *Journal of Magnetic Resonance Imaging* 2001; **13**(4)**:** 534-546.

24. Takeuchi H, Taki Y, Hashizume H, Asano K, Asano M, Sassa Y *et al.* Impact of videogame play on the brain's microstructural properties: Cross-sectional and longitudinal analyses. *Mol Psychiatry* 2016; **21:** 1781-1789.

25. Takeuchi H, Taki Y, Hashizume H, Sassa Y, Nagase T, Nouchi R *et al.* Failing to deactivate: the association between brain activity during a working memory task and creativity. *Neuroimage* 2011; **55**(2)**:** 681-687.

26. Hugenschmidt CE, Peiffer AM, Kraft RA, Casanova R, Deibler AR, Burdette JH *et al.* Relating imaging indices of white matter integrity and volume in healthy older adults. *Cerebral Cortex* 2008; **18**(2)**:** 433-442.

27. Smith SM, Jenkinson M, Johansen-Berg H, Rueckert D, Nichols TE, Mackay CE *et al.* Tract-based spatial statistics: voxelwise analysis of multi-subject diffusion data. *Neuroimage* 2006; **31**(4)**:** 1487-1505.
